# Association of clonal hematopoiesis of indeterminate potential with cardiovascular mortality in cardiovascular-kidney-metabolic syndrome: a large prospective cohort study

**DOI:** 10.3389/fendo.2026.1910361

**Published:** 2026-10-07

**Authors:** Zehao Zhao, Changsheng Ma, Libo Zhao, Xiliang Zhao, Quan Li, Yaodong Ding, Yang Zhang, Li Lin, Wenjie Wang, Jiayi Han, Yingxuan Bai, Tianyi Wang, Yong Zeng

**Affiliations:** 1 Department of Cardiology, Beijing Anzhen Hospital, Capital Medical University, Beijing, China; 2 National Clinical Research Center For Cardiovascular Disease, Beijing, China; 3 Beijing Institute of Heart Lung and Blood Vessel Diseases, Beijing, China

**Keywords:** cardiovascular mortality, cardiovascular-kidney-metabolic syndrome, clonal hematopoiesis of indeterminate potential, cohort study, proteomics

## Abstract

**Background:**

Cardiovascular-kidney-metabolic (CKM) syndrome represents a progressive pathophysiological continuum. While clonal hematopoiesis of indeterminate potential (CHIP) is known to increase the risk of CKM disease onset and adverse prognoses, its overall and stage-specific impacts on cardiovascular mortality within the CKM framework, alongside the circulating proteomic mechanisms mediating this excess risk, remain largely unknown.

**Methods:**

Participants from the UK Biobank with available CHIP data across the CKM syndrome continuum were included. The primary outcome was cardiovascular mortality. Kaplan-Meier curves and multivariable Cox proportional hazards models were utilized to evaluate the incremental risk conferred by CHIP. Sensitivity and subgroup analyses were conducted to ascertain result robustness. Within UKB Pharma Proteomics Project (UKB-PPP), circulating proteins jointly associated with CHIP and cardiovascular mortality were identified, followed by functional enrichment and mediation analyses to elucidate the underlying mechanisms.

**Results:**

During a median follow-up of 16.5 years, 6,515 cardiovascular deaths were recorded among 288,384 participants across CKM stages 1–4. Across all varying CKM stages, individuals harboring Large CHIP exhibited a significantly higher risk of cardiovascular mortality compared to the non-CHIP group (all log-rank P < 0.05). Following multivariable adjustment, Large CHIP emerged as an independent risk factor for cardiovascular mortality (hazard ratio, 1.24; 95% confidence interval, 1.09–1.40), with subgroup analyses confirming consistent effects regardless of CKM stage or established cardiometabolic and renal diseases. Proteomic profiling, corroborated by UKB-PPP, revealed that the mechanisms driving this excess risk predominantly involve immune-inflammatory dysregulation and tissue structural remodeling. This pathway was highlighted by critical downstream mediators, including the structural component DSC2 (mediating 34.3% of the risk) and key immune-regulatory receptors such as VSIG4 (33.1%) and CD300C (29.2%).

**Conclusions:**

CHIP is an independent risk factor for cardiovascular mortality across all stages of CKM syndrome. Mechanistically, CHIP exacerbates this adverse outcome primarily by driving systemic inflammation, immune dysregulation, and aberrant tissue remodeling.

## Graphical Abstract

**Figure:**
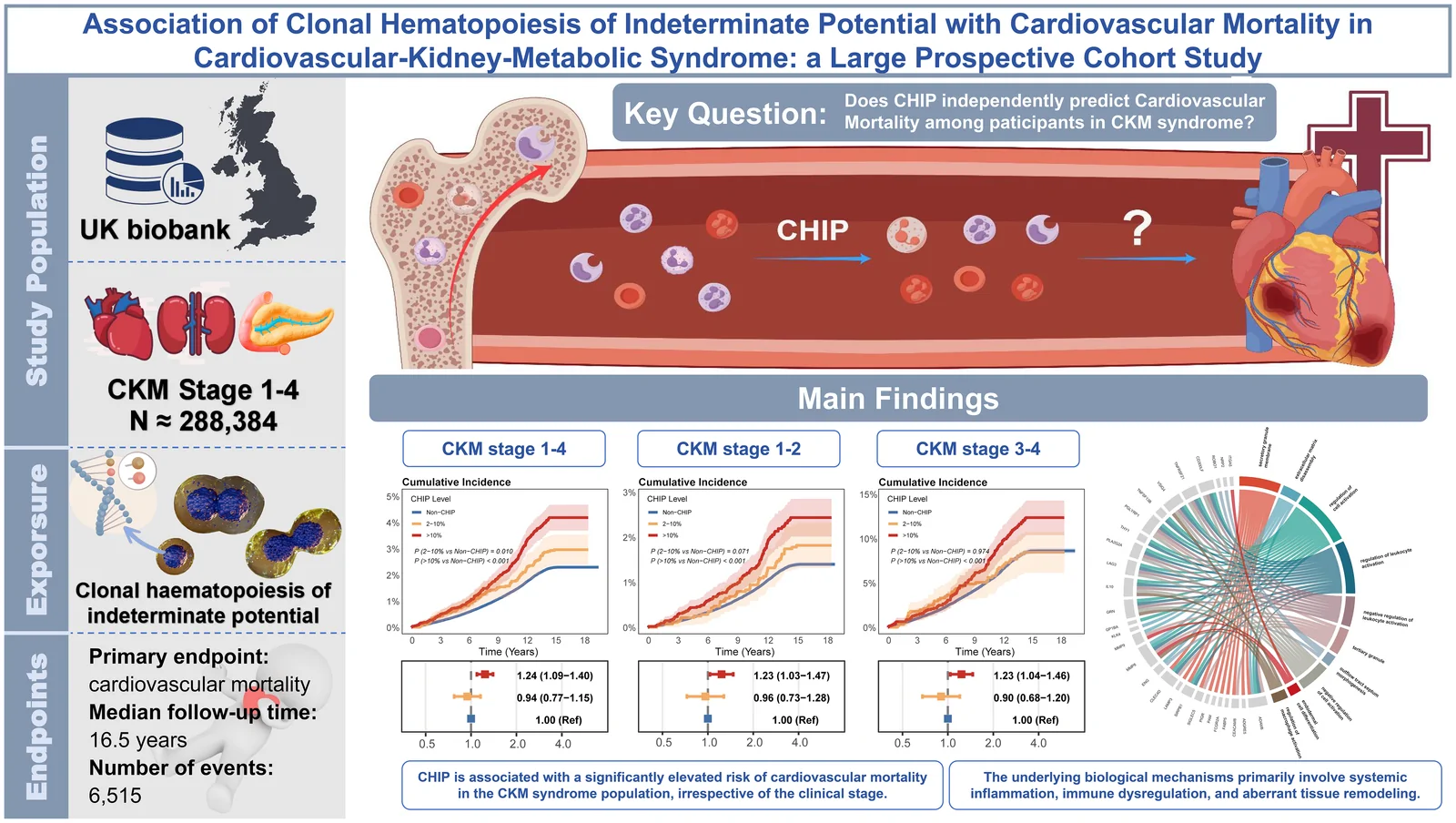

## 1 Introduction

Cardiovascular, kidney, and metabolic diseases frequently present concomitantly or sequentially in clinical practice due to shared risk factors and overlapping pathophysiological mechanisms. In 2023, the American Heart Association (AHA) introduced the conceptual framework of Cardiovascular-Kidney-Metabolic (CKM) syndrome (1). This classification redefines obesity, diabetes, chronic kidney disease, and cardiovascular disease not as isolated clinical entities, but as interconnected manifestations of a unified systemic process across different organs and disease stages. This framework advances the timing of clinical intervention and substantially broadens the identified at-risk population, extending from individuals with established disease (Stages 3 to 4) to those with early-stage risk factors (Stages 1 to 2). Epidemiological data indicate that approximately 90% of adults in the United States fall within CKM stages 1 to 4 (2). Although current clinical tools estimate the risk of CKM progression and incident cardiovascular events, the specific biological determinants driving fatal outcomes, particularly cardiovascular mortality, among patients across the CKM spectrum require further investigation (3, 4). Given that systemic inflammation and immune dysregulation constitute the core pathological foundation of the CKM continuum, identifying novel upstream drivers of aberrant inflammation is essential.

Clonal hematopoiesis of indeterminate potential (CHIP) refers to the somatic clonal expansion of hematopoietic stem cells resulting from acquired driver mutations (5). Clinically, CHIP is defined by the presence of somatic mutations in myeloid-associated driver genes with a variant allele frequency of ≥ 2%, occurring in the absence of unexplained cytopenias or morphological evidence of hematological dysplasia (6). The prevalence of CHIP is highly age-dependent, reaching 10% to 20% among individuals older than 70 years (7). Mechanistically, mutations in common driver genes (such as TET2 and DNMT3A) propagate pro-inflammatory leukocyte phenotypes, initiating systemic immune dysregulation that directly intersects with the pathological mechanisms of CKM syndrome.

(3-1)Historically recognized as a precursor to hematological malignancies, CHIP has been shown to increase all-cause mortality, with early population studies predominantly highlighting the excess risk driven by hematological cancer progression (Genovese et al.) (8). However, subsequent seminal analyses clarified that a substantial proportion of this excess mortality is heavily driven by non-oncological cardiovascular deaths (Jaiswal et al.) (7).

Regarding disease incidence, emerging epidemiological evidence has linked the presence of CHIP to the onset of isolated metabolic, renal, and specific cardiovascular disorders (9–11). Nevertheless, population-scale investigations evaluating individual cardiovascular endpoints have revealed notable outcome-specific heterogeneity. For instance, recent UK Biobank analyses demonstrated that CHIP is significantly associated with an increased risk of incident heart failure and atrial fibrillation, whereas its associations with incident atherosclerotic cardiovascular disease (ASCVD) and stroke were less pronounced or null (Kar et al., 2022). Beyond disease onset, CHIP status has also been implicated in adverse prognoses and secondary mortality among cohorts with pre-existing individual conditions (12).

However, prior studies have largely evaluated these disorders as isolated entities, leaving the prognostic impact of CHIP across the interconnected CKM spectrum unexplored. Specifically, whether CHIP acts as a uniform or stage-specific driver of cardiovascular mortality across early (Stages 1–2) and advanced (Stages 3–4) CKM continuum remains unknown. Determining these associations and their underlying biological mechanisms is essential to refine risk stratification and guide targeted therapies in CKM syndrome.

## 2 Methods

### 2.1 Study design

This is a large-scale, prospective cohort study based on the UKB. The study design and the reporting of results strictly adhered to the Strengthening the Reporting of Observational Studies in Epidemiology (STROBE) reporting guidelines (13).

### 2.2 Study participants

The UKB recruited approximately 500,000 individuals aged 40–69 years across England, Scotland, and Wales between 2006 and 2010. During the baseline assessment, comprehensive data on sociodemographic profiles, lifestyle behaviors, and clinical history were systematically recorded alongside standardized physical examinations. Peripheral blood samples were obtained for systemic biomarker quantification, microarray genotyping, and whole-exome sequencing (WES). The stage of CKM syndrome was defined according to the recent presidential advisory from the American Heart Association (AHA) (1, 4). For the primary stratified analyses, CKM stages 3 and 4 were combined to define an advanced CKM group and to facilitate comparison with the early CKM group comprising stages 1–2. This analytical grouping was used to improve statistical stability and was not intended to imply clinical equivalence between CKM stages 3 and 4. The present analysis included participants from the UKB who had available sequencing data for CHIP. Subsequently, individuals were excluded from the study if they met any of the following criteria: (1) withdrawal of informed consent or loss to follow-up; (2) a baseline diagnosis of hematologic or lymphoid malignancies; or (3) failure to pass genomic quality control (QC) thresholds. Baseline characteristics were described for the overall study cohort of 443,708 participants. Among them, 346,405 participants had sufficient information to be assigned a CKM stage according to the American Heart Association definition. The remaining 39,282 participants were excluded from CKM-stage-specific analyses because CKM staging was missing or could not be determined. CKM stage 0 was excluded from the primary analysis because it represents individuals without identifiable CKM risk factors or established cardiovascular, kidney, or metabolic disease. The primary analysis was therefore restricted to CKM stages 1–4 to evaluate the prognostic role of CHIP across the CKM continuum. **Figure 1** presents the inclusion and exclusion process of the study participants. Current study received formal approval from the North West Multi-centre Research Ethics Committee (Application Number 103736). All participants provided electronic informed consent before data collection and clinical assessment.

**Figure 1:**
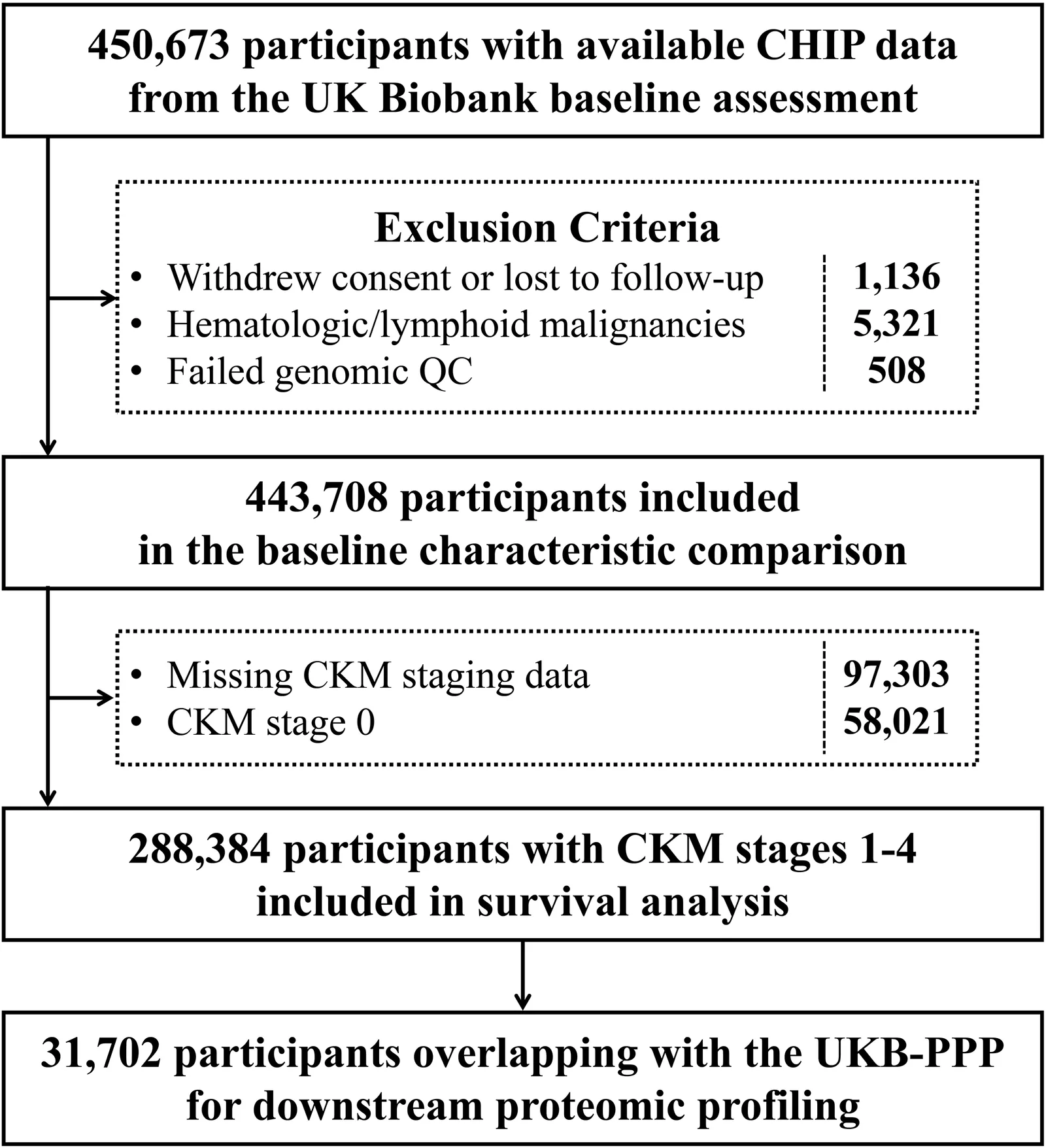
Flowchart of study participant selection.

### 2.3 Exposures

CHIP was ascertained from whole blood-derived WES. For the current study, these data were directly retrieved from the UKB database: (1) Data-Fields 30105 (number of CHIP variants), 30106 (CHIP variant nomenclature), and 30107 (variant allele frequency [VAF]). These somatic mutations were identified using a previously validated bioinformatic pipeline (14). The primary exposures of interest were defined as the presence of any CHIP (defined by a VAF ≥ 2%) and large CHIP (defined by a VAF ≥ 10%) (12, 15). Furthermore, to investigate gene-specific effects, we independently evaluated the CHIP subtypes driven by the three most prevalent mutant genes: DNMT3A, TET2, and ASXL1. Comprehensive details regarding the sequencing, variant calling, and quality control procedures are provided in the **Supplementary Methods**.

### 2.4 Other covariates

Covariates encompassed demographic, socioeconomic, behavioral, and clinical factors. Demographic variables included age, sex, and ethnicity. Socioeconomic status included annual household income (<£18,000, £18,000-£30,999, £31,000-£51,999, £52,000-£100,000, and >£100,000), educational attainment (16) (categorized as university degree or equivalent, high school or equivalent, middle school or equivalent, and primary school or below), and employment status (employed, unemployed, or retired).

Lifestyle factors were ascertained via self-report. Smoking status was classified as never, former, or current. Alcohol consumption was categorized into never, former, safe, or dangerous drinking levels based on reported frequency and volume (17). Physical activity was stratified into low, moderate, and high levels. Sleep health, determined by sleep duration, was classified as unhealthy, intermediate, or healthy. Baseline clinical and biochemical measurements incorporated body mass index (BMI), systolic blood pressure (SBP), total cholesterol (TC), triglycerides (TG), C-reactive protein (CRP), and glycated hemoglobin (HbA1c). Comprehensive definitions and specific data provenance for all covariates are detailed in the **Supplementary Methods** (17, 18).

Missing values for covariates were addressed using a random forest-based iterative imputation approach.

### 2.5 Blood proteomic profiling

Proteomic analyses were conducted within a predefined sub-cohort comprising 31,702 participants. Baseline plasma proteome profiles were characterized utilizing data from the UKB Pharma Proteomics Project (UKB-PPP). Briefly, venous blood samples were collected in EDTA-coated tubes, centrifuged (2500g, 10 min, 4 °C) to isolate plasma, aliquoted, and subsequently stored at −80 °C. Proteomic quantification was performed by Olink Analytical Services (Uppsala, Sweden) using the antibody-based Olink Explore 3072 Proximity Extension Assay (PEA) technology. This platform captured 2,923 unique proteins across four comprehensive panels: cardiometabolic, inflammation, neurology, and oncology.

Protein levels were reported as Normalized Protein Expression (NPX) values, which are on a relative log2 scale. Stringent QC procedures were implemented prior to data release, with both intra- and inter-plate coefficients of variation consistently below 10% and 20%, respectively. Comprehensive details regarding the Olink PEA assay, sample processing, and overarching QC metrics are extensively documented in prior publications and the official companion document. In the present study, to ensure data robustness, protein features with a missingness rate exceeding 30% were excluded from the primary analyses, resulting in a final analytical set of 2,920 unique proteins. Missing proteomic data were handled using a constant down-shift imputation method to account for values below the detection limit. For each protein, missing values were replaced by its minimum observed value minus 0.5 standard deviations.

### 2.6 Ascertainment of the clinical outcome

The primary endpoint of this study was defined exclusively as cardiovascular mortality. The occurrence of this endpoint was ascertained through linkage to national death registries, specifically the NHS Information Centre for England and Wales, and the NHS Central Register for Scotland. Cardiovascular mortality was strictly identified when the underlying primary cause of death was coded according to the International Classification of Diseases, Tenth Revision (ICD-10) with a code ranging from I00 to I99. In sensitivity analyses, this definition was expanded to include any death record where these codes were documented as either the primary or a contributory cause. For the time-to-event analyses, person-years of follow-up were calculated from the date of the baseline assessment until the date of cardiovascular death, the date of death from other competing causes, the date of withdrawal from the cohort, or the administrative censoring date (December 31, 2022), whichever occurred first.

### 2.7 Statistical analysis

All statistical tests were two-sided, and a p value < 0.05 was considered statistically significant. All analyses were performed using R statistical software (version 4.4.3; The R Foundation for Statistical Computing, Vienna, Austria).

#### 2.7.1 Baseline characteristics

Continuous variables are presented as means with standard deviations (SD) for normally distributed data, or as medians with interquartile ranges (IQR) for skewed data. Categorical variables are expressed as frequencies and percentages. Comparisons across groups for continuous variables were conducted using one-way analysis of variance (ANOVA) or the Kruskal-Wallis H test, as appropriate based on data distribution. Categorical variables were compared using Pearson’s chi-square test.

#### 2.7.2 Survival analyses

Kaplan-Meier survival curves were generated to visualize the cumulative risk of cardiovascular mortality stratified by CHIP status, with inter-group differences evaluated using the log-rank test. Multivariable Cox proportional hazards regression models were subsequently constructed to quantify the independent prognostic impact of CHIP on cardiovascular risk. Effect sizes are reported as hazard ratios (HRs) with corresponding 95% confidence intervals (CIs). The proportional hazards assumption was verified for all Cox regression models using Schoenfeld residuals, and no significant violations were observed.

To ensure the robustness of the primary findings, several predefined sensitivity analyses were performed. First, to mitigate the potential bias of reverse causality, participants who experienced the endpoint within the first two years of follow-up were excluded from the analysis. Second, to account for competing risks, analyses were re-evaluated using a competing risk model, with deaths from other causes treated as competing events. Third, models were re-evaluated using the expanded sensitivity definition for cardiovascular mortality, which incorporated contributory causes of death. Furthermore, prespecified subgroup analyses were performed to assess the consistency of the association across CKM stage and major baseline demographic and clinical subgroups.

#### 2.7.3 Proteomics-based mechanistic analysis

Multivariable logistic regression and Cox proportional hazards models were employed to identify circulating proteins associated with any CHIP and incident cardiovascular mortality, respectively. These models were adjusted for age, sex, ethnicity, Townsend Deprivation Index, household income, education level, employment status, smoking status, alcohol consumption, physical activity level, sleep habits, C-reactive protein, triglycerides, glycated hemoglobin, body mass index, systolic blood pressure, and total cholesterol. Proteins associated with CHIP status and cardiovascular mortality were first identified separately using multivariable-adjusted models; the final set of overlapping proteins was defined as the intersection of proteins that were significant in both analyses. For the identified significant proteins, downstream functional enrichment analysis was conducted using the Metascape platform to elucidate the implicated biological mechanisms (19). This functional annotation encompassed Kyoto Encyclopedia of Genes and Genomes (KEGG) pathways alongside comprehensive Gene Ontology (GO) categories (comprising biological processes, cellular components, and molecular functions) (20, 21). To ensure rigorous statistical scaling, the genes encoding the 2,920 analyzed plasma proteins were designated as the custom background reference set. Stringent significance thresholds were strictly enforced: only pathways and terms demonstrating a P-value < 0.01, a minimum overlap of 3 gene counts, and an enrichment factor > 1.5 (calculated as the ratio of observed to expected gene counts) were retained. Finally, to minimize redundancy, the resultant significant terms were hierarchically clustered into representative network nodes based on their biological membership similarities.

Subsequent causal mediation analyses were applied to identify intermediate proteins driving the CHIP-related excess risk of cardiovascular mortality, employing single-mediator models to evaluate each candidate protein independently. Specifically, the regmedint package in R was utilized to accommodate the time-to-event outcomes (Cox proportional hazards models) and continuous mediators (linear regression models). We estimated the pure natural direct effect (PNDE) and pure natural indirect effect (PNIE), and calculated the proportion of the effect mediated (PM) on the original scale. To account for multiple comparisons across the candidate proteins, P values for the indirect effects were adjusted using the Benjamini-Hochberg False Discovery Rate (FDR) procedure. An FDR-adjusted P value < 0.05 was considered statistically significant.

## 3 Results

### 3.1 Baseline characteristics

**Table 1** summarizes the baseline clinical and demographic characteristics of the 443,708 eligible participants (mean age, 56.48 ± 8.10 years; 45.6% male) with available CHIP sequencing data, stratified by CHIP variant allele frequency (VAF) level (non-CHIP, small CHIP [2–10%), and large CHIP [≥10%]). Among them, 15,038 individuals (3.39%) harbored at least one CHIP mutation, comprising 5,182 individuals with small CHIP and 9,856 with large CHIP. Compared with non-carriers, CHIP carriers were progressively older (mean age: 56.34 ± 8.10 years for non-CHIP vs. 60.26 ± 6.75 years for small CHIP and 60.91 ± 6.64 years for large CHIP; P < 0.001). Furthermore, the CHIP cohort exhibited a more socioeconomically disadvantaged profile, characterized by lower household income, reduced educational attainment, and differential employment status (all P < 0.001), whereas the Townsend deprivation index showed no significant variation (P = 0.598). Lifestyle and clinical assessments indicated that CHIP carriers were more likely to have a history of smoking (P < 0.001) and presented with altered cardiometabolic profiles, including elevated glycated hemoglobin (HbA1c; P < 0.001) and systolic blood pressure (SBP; P < 0.001), while body mass index (BMI) remained comparable across groups (P = 0.108). Consistent with these findings, CHIP carriers—particularly those with large CHIP—bore a substantially higher burden of cardiovascular-kidney-metabolic (CKM) dysfunction, as evidenced by elevated PREVENT equation scores and a higher prevalence of overt CKM diseases and advanced CKM stages (all P < 0.001). The prevalence of overall CHIP and specific driver mutations across the spectrum of CKM stages is detailed in **Supplementary Table 1**; **Figure 2**. Notably, the prevalence of the most frequent driver mutations, including DNMT3A, TET2, and ASXL1, exhibited a clear incremental trend as CKM syndrome progressed from early to advanced stages.

**Table 1:** Baseline characteristics of the study population stratified by the presence or absence of CHIP.

	Level	Overall	>10%	2-10%	Non-CHIP	p	SMD
n		443708	9856	5182	428670		
Age, years, mean (SD)		56.48 (8.10)	60.91 (6.64)	60.26 (6.75)	56.34 (8.10)	<0.001	0.414
Sex, n (%)	Female	241505 (54.4)	5216 (52.9)	2848 (55.0)	233441 (54.5)	0.008	0.027
	Male	202203 (45.6)	4640 (47.1)	2334 (45.0)	195229 (45.5)		
Ethnicity, n (%)	non-White	19614 (4.5)	313 (3.2)	181 (3.5)	19120 (4.5)	<0.001	0.045
	White	418010 (95.5)	9428 (96.8)	4935 (96.5)	403647 (95.5)		
Townsend Deprivation Index, median [IQR]		-2.15 [-3.65, 0.52]	-2.15 [-3.62, 0.56]	-2.21 [-3.65, 0.49]	-2.15 [-3.65, 0.52]	0.598	0.014
Household Income, n (%)	Less than 18,000	85802 (22.6)	2410 (29.3)	1201 (27.8)	82191 (22.4)	<0.001	0.157
	18,000 to 30,999	96341 (25.4)	2400 (29.2)	1177 (27.3)	92764 (25.3)		
	31,000 to 51,999	98997 (26.1)	1891 (23.0)	1041 (24.1)	96065 (26.2)		
	52,000 to 100,000	77424 (20.4)	1199 (14.6)	706 (16.4)	75519 (20.6)		
	Greater than 100,000	20603 (5.4)	320 (3.9)	189 (4.4)	20094 (5.5)		
Education Level, n (%)	College education	211806 (48.3)	4077 (41.9)	2281 (44.6)	205448 (48.5)	<0.001	0.117
	High school	77086 (17.6)	1724 (17.7)	891 (17.4)	74471 (17.6)		
	Top school	74188 (16.9)	1636 (16.8)	871 (17.0)	71681 (16.9)		
	Primary school or less	75285 (17.2)	2287 (23.5)	1071 (20.9)	71927 (17.0)		
Employment Status, n (%)	Employed	257059 (58.5)	4024 (41.2)	2320 (45.1)	250715 (59.1)	<0.001	0.269
	Unemployed	35932 (8.2)	645 (6.6)	345 (6.7)	34942 (8.2)		
	Retired	146098 (33.3)	5095 (52.2)	2475 (48.2)	138528 (32.7)		
Smoking Status, n (%)	Never	241964 (54.8)	4621 (47.2)	2514 (48.9)	234829 (55.1)	<0.001	0.107
	Previous	153006 (34.7)	3924 (40.1)	2022 (39.3)	147060 (34.5)		
	Current	46468 (10.5)	1250 (12.8)	604 (11.8)	44614 (10.5)		
Alcohol Intake, n (%)	Never	19473 (5.3)	430 (5.2)	224 (5.2)	18819 (5.3)	0.015	0.035
	Previous	15784 (4.3)	373 (4.5)	207 (4.8)	15204 (4.3)		
	Safe	174840 (47.4)	3752 (45.5)	2020 (47.1)	169068 (47.4)		
	Dangerous	158903 (43.1)	3683 (44.7)	1840 (42.9)	153380 (43.0)		
Physical Activity, n (%)	low	63494 (18.5)	1325 (17.9)	700 (17.9)	61469 (18.5)	0.481	0.013
	moderate	139525 (40.6)	3050 (41.2)	1621 (41.4)	134854 (40.6)		
	high	140344 (40.9)	3021 (40.8)	1596 (40.7)	135727 (40.9)		
Sleep Habits, n (%)	Unhealthy	32235 (7.3)	738 (7.6)	407 (7.9)	31090 (7.3)	0.001	0.035
	Medium	110099 (25.0)	2594 (26.5)	1267 (24.7)	106238 (25.0)		
	Healthy	298043 (67.7)	6440 (65.9)	3464 (67.4)	288139 (67.7)		
CRP, mg/L, (median [IQR])		1.32 [0.65, 2.75]	1.45 [0.73, 3.00]	1.43 [0.72, 2.95]	1.32 [0.65, 2.74]	<0.001	0.038
TG, mmol/L, (median [IQR])		1.48 [1.04, 2.15]	1.53 [1.08, 2.19]	1.52 [1.07, 2.14]	1.48 [1.04, 2.15]	<0.001	0.018
HbA1c, %, (mean (SD))		5.46 (0.62)	5.52 (0.63)	5.52 (0.63)	5.45 (0.62)	<0.001	0.073
BMI, kg/m2, (mean (SD))		27.41 (4.77)	27.49 (4.61)	27.50 (4.70)	27.41 (4.77)	0.108	0.012
SBP, mmHg, (mean (SD))		137.78 (18.61)	141.37 (18.87)	140.94 (19.03)	137.66 (18.58)	<0.001	0.132
TC, mmol/L, (mean (SD))		5.70 (1.14)	5.65 (1.19)	5.70 (1.18)	5.70 (1.14)	<0.001	0.03
PREVENT Equation Score, (median [IQR])		0.06 [0.03, 0.10]	0.09 [0.05, 0.12]	0.08 [0.05, 0.12]	0.06 [0.03, 0.10]	<0.001	0.3
CVD, n(%)		35926 (8.1)	1141 (11.6)	558 (10.8)	34227 (8.0)	<0.001	0.081
IHD, n(%)		24575 (5.5)	770 (7.8)	389 (7.5)	23416 (5.5)	<0.001	0.063
HF, n(%)		2394 (0.5)	94 (1.0)	38 (0.7)	2262 (0.5)	<0.001	0.033
AF, n(%)		7353 (1.7)	232 (2.4)	102 (2.0)	7019 (1.6)	<0.001	0.034
Stroke, n(%)		7991 (1.8)	261 (2.6)	134 (2.6)	7596 (1.8)	<0.001	0.04
CKD, n(%)		5664 (1.3)	209 (2.1)	93 (1.8)	5362 (1.3)	<0.001	0.045
T2DM, n(%)		11922 (2.7)	387 (3.9)	165 (3.2)	11370 (2.7)	<0.001	0.048
CKM stage, n(%)	stage 0	58021 (16.7)	981 (12.6)	608 (15.0)	56432 (16.9)	<0.001	0.14
	stage 1	50418 (14.6)	978 (12.5)	490 (12.1)	48950 (14.6)		
	stage 2	199950 (57.7)	4583 (58.7)	2346 (57.7)	193021 (57.7)		
	stage 3-4	38016 (11.0)	1271 (16.3)	619 (15.2)	36126 (10.8)		

AF, atrial fibrillation; BMI, body mass index; CHIP, clonal hematopoiesis of indeterminate potential; CI, confidence interval; CKD, chronic kidney disease; CKM, cardiovascular-kidney-metabolic; CRP, C-reactive protein; CVD, cardiovascular disease; HbA1c, glycated hemoglobin; HF, heart failure; HR, hazard ratio; IHD, ischemic heart disease; IQR, interquartile range; MET, metabolic equivalent of task; PREVENT, Predicting Risk of cardiovascular disease EVENTs; SBP, systolic blood pressure; SD, standard deviation; SMD, standardized mean difference; T2DM, type 2 diabetes mellitus; TC, total cholesterol; TDI, Townsend deprivation index; TG, triglycerides.

**Figure 2:**
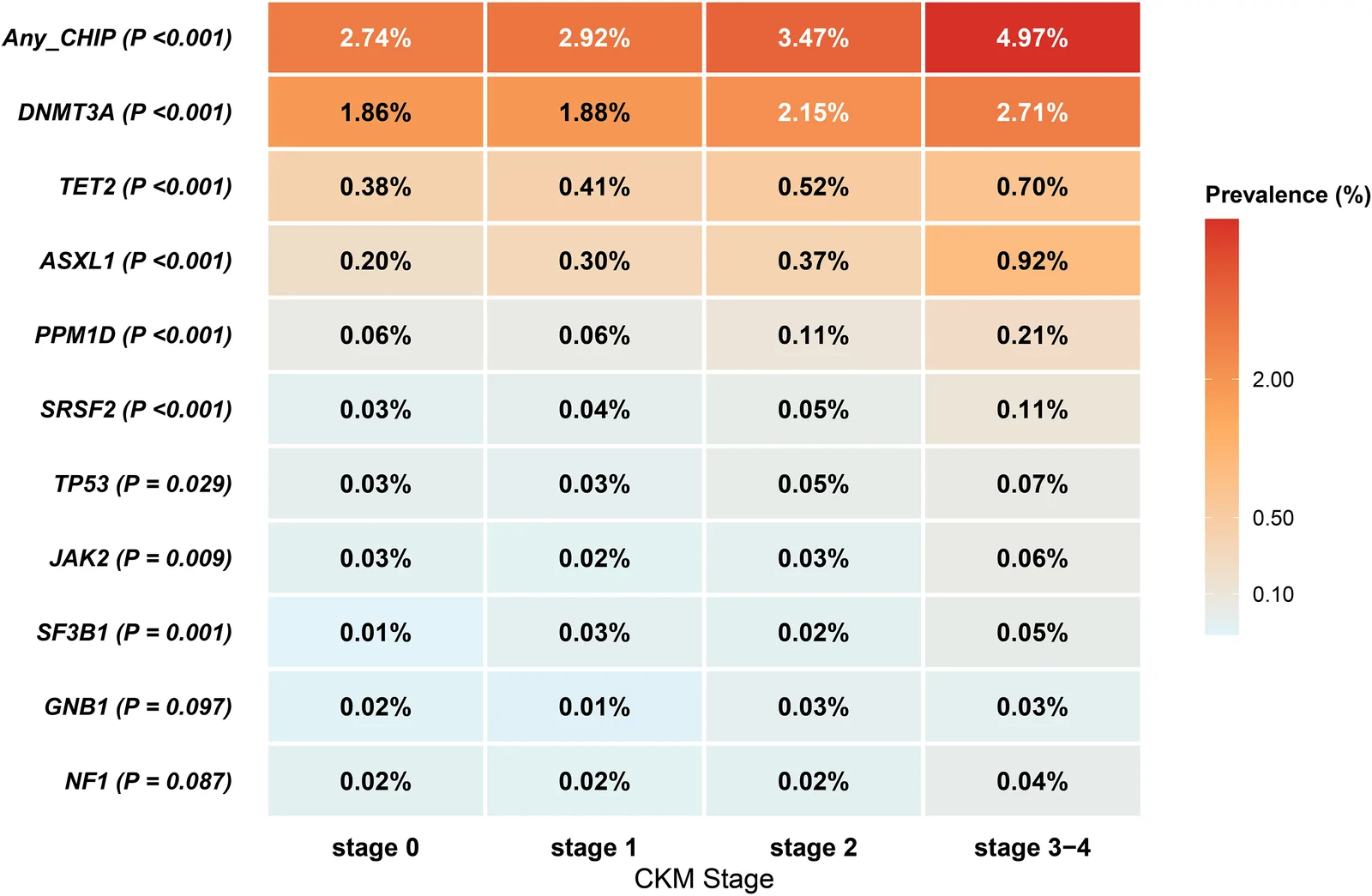
Prevalence of the top 10 most frequent CHIP mutations across CKM syndrome stages.

### 3.2 Association of CHIP and gene-specific CHIP subtypes with cardiovascular mortality

Over a median follow-up of 16.5 years, 6,515 cardiovascular deaths were documented among the 288,384 participants with CKM stages 1-4. Kaplan-Meier survival estimates revealed that harboring large CHIP was associated with a substantially higher cumulative risk of cardiovascular mortality compared with non-carriers, a adverse association observed consistently irrespective of baseline CKM stage (log-rank P < 0.001; **Figure 3A**). To delineate the prognostic implications of specific driver genes, the analysis was extended to the three most prevalent large CHIP mutations (DNMT3A, TET2, and ASXL1). Cumulative incidence trajectories (**Figures 3B–D**) demonstrated that this excess mortality risk was not solely linked to a singular mutational profile. Rather, the presence of any of these three predominant large CHIP clones was associated with a markedly elevated risk of cardiovascular death. Importantly, this adverse prognostic penalty remained robust both in the overall cohort and across distinct CKM strata. Furthermore, detailed time-specific cumulative risks and corresponding within-CKM group comparison p-values across all evaluation intervals are comprehensively provided in **Supplementary Table 4**.

**Figure 3:**
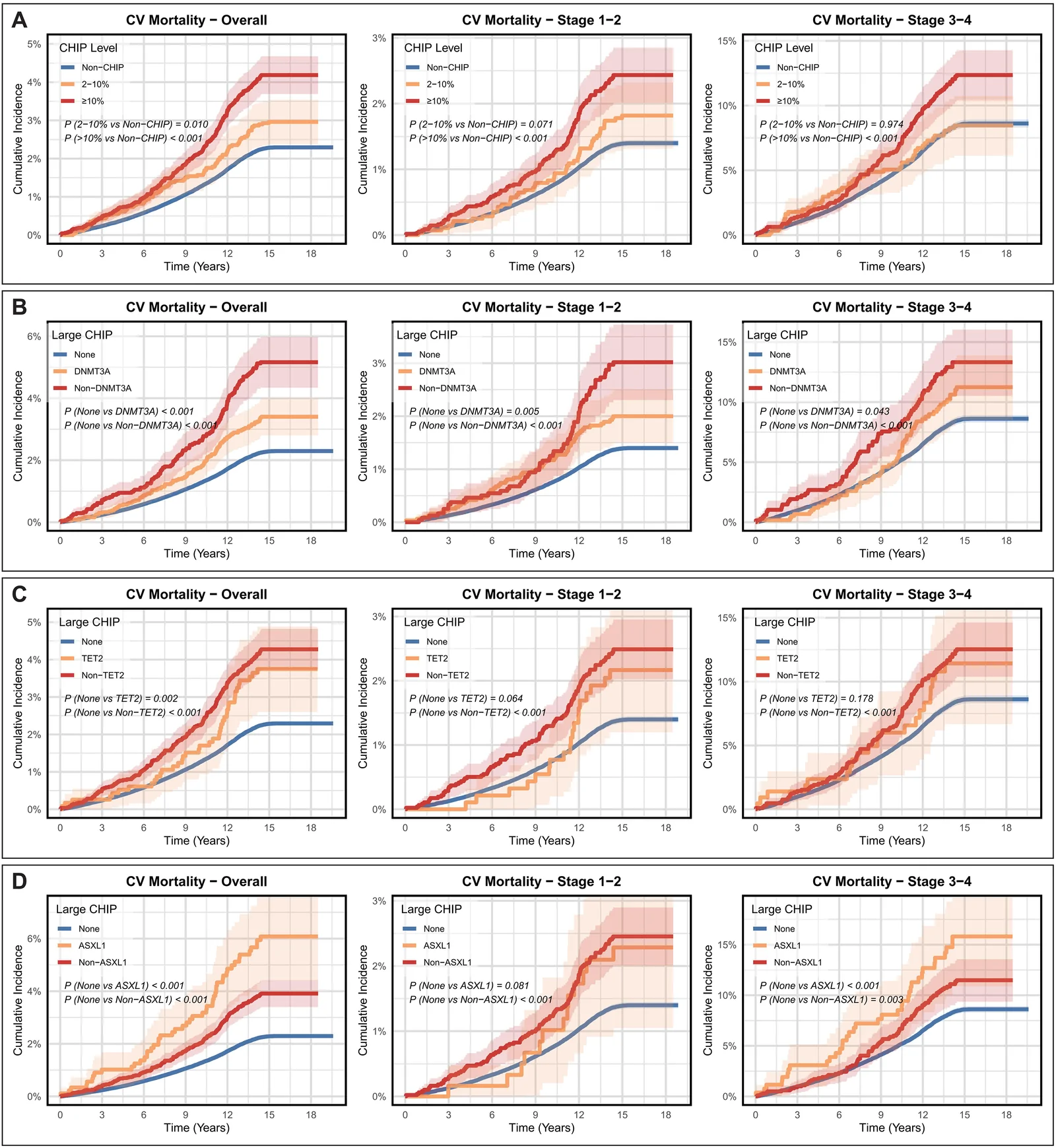
Cumulative incidence of cardiovascular mortality stratified by CHIP status across different stages of CKM syndrome. **(A)** Cumulative incidence of cardiovascular mortality categorized by overall CHIP VAF levels: Non-CHIP, VAF 2–10%, and VAF ≥10%. **(B–D)** Cumulative incidence of CV mortality stratified by gene-specific Large CHIP (defined as VAF ≥10%) for DNMT3A **(B)**, TET2 **(C)**, and ASXL1 **(D)**. For all panels, plots from left to right represent the overall study population, participants in CKM stages 1–2, and participants in CKM stages 3–4, respectively. P-values indicate the statistical significance of differences between the specified CHIP groups and the reference group, calculated using log-rank test. CHIP, clonal hematopoiesis of indeterminate potential; CKM, cardiovascular-kidney-metabolic.

To ascertain the independent prognostic value of large CHIP for cardiovascular mortality, multivariable Cox proportional hazards regression models were constructed. The models were hierarchically adjusted for demographic characteristics, socioeconomic status, behavioral factors, and clinical and biochemical indices (Models 0 through 4). As illustrated in **Figure 4A**, overall large CHIP, encompassing mutations across any driver gene, emerged as a robust and independent predictor of cardiovascular death, retaining statistical significance even in the fully adjusted Model 4 (HR and 95% CI: 1.24 [1.09−1.40]). Importantly, this independent prognostic association was consistently maintained across the entire cohort, as well as within both the early (Stages 1-2, HR and 95% CI: 1.23 [1.03−1.47]) and advanced (Stages 3-4, HR and 95% CI: 1.23 [1.04−1.46]) CKM strata. These primary findings were further corroborated by the highly consistent results depicted in **Supplementary Figures S1**, **S3**.

**Figure 4:**
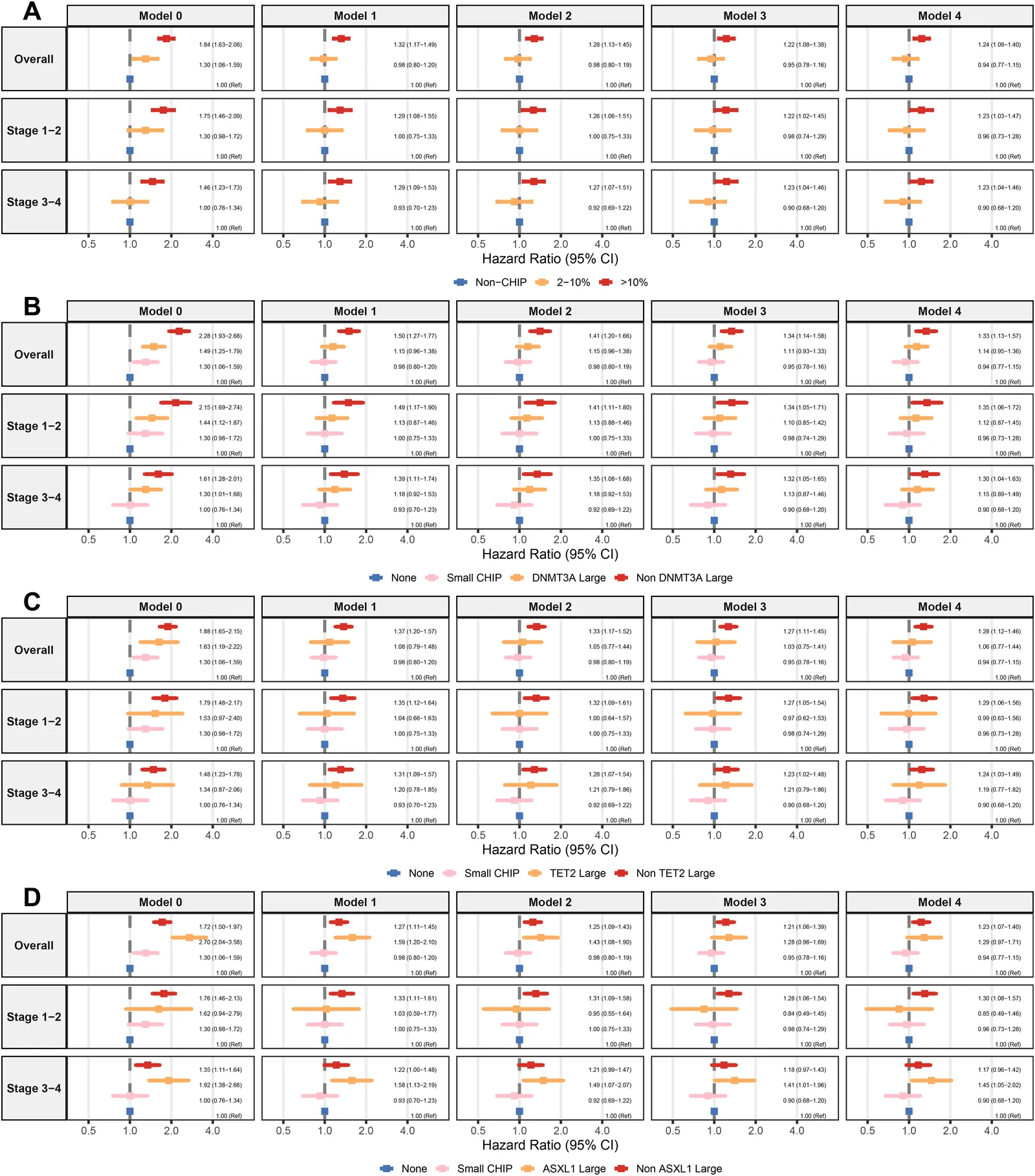
Association of overall and gene-specific CHIP mutations with the risk of cardiovascular mortality across different CKM stages. **(A)** HRs and 95% CIs for cardiovascular mortality categorized by overall CHIP VAF levels: Non-CHIP, 2–10%, and ≥10%. **(B–D)** HRs and 95% CIs for cardiovascular mortality stratified by gene-specific CHIP status for DNMT3A **(B)**, TET2 **(C)**, and ASXL1 **(D)**. Analyses were performed in the overall study population and stratified by CKM stages (Stage 1–2 and Stage 3–4). Risk estimates were assessed using Cox proportional hazards models with progressive adjustment for potential confounders. Model 0: Unadjusted. Model 1: Adjusted for age, sex, and race. Model 2: Adjusted for Model 1 covariates plus education level, employment status, income level, and TDI. Model 3: Adjusted for Model 2 covariates plus smoking status, alcohol consumption, physical activity, and sleep status. Model 4: Adjusted for Model 3 covariates plus C-reactive protein, triglycerides, glycated hemoglobin, body mass index, systolic blood pressure, and total cholesterol. CI, confidence interval; CHIP, clonal hematopoiesis of indeterminate potential; CKM, cardiovascular-kidney-metabolic.

Gene-specific analyses yielded a divergent pattern. The initial associations between cardiovascular mortality and isolated large clones of DNMT3A, TET2, and ASXL1 (**Figures 4B–D**) were sharply attenuated immediately upon adjustment for demographic factors (Model 1, primarily driven by age, sex, and race), with subsequent hierarchical adjustments exhibiting minimal further influence. Consequently, the prognostic impacts of these single-gene large CHIP mutations were rendered non-significant within the general cohort. Despite this overall attenuation, subgroup stratification revealed that large ASXL1 CHIP was independently associated with cardiovascular mortality exclusively among participants in CKM stages 3-4. However, given the limited sample sizes within specific gene-strata, this exploratory observation should be interpreted with caution, as gene-specific analyses remain inherently underpowered to establish definitive variant-specific prognostic hierarchies.

### 3.3 Subgroup analyses

Subgroup analyses (**Figure 5**) further demonstrated that the prognostic association between large CHIP and cardiovascular mortality remained robust and consistent across both early and advanced CKM stages. Furthermore, this detrimental impact was homogeneous irrespective of prevalent baseline comorbidities, including CVD, CKD, and T2DM (all P for interaction > 0.05). Notably, the association was attenuated and became statistically non-significant among specific demographic and clinical subgroups, including younger individuals (≤ 60 years), current smokers, never-drinkers, highly physically active participants, and those with a normal baseline BMI, hypertriglyceridemia, or hypercholesterolemia. However, with the explicit exception of hypertriglyceridemia (P for interaction = 0.043), formal interaction testing did not reveal significant effect modification by these variables.

**Figure 5:**
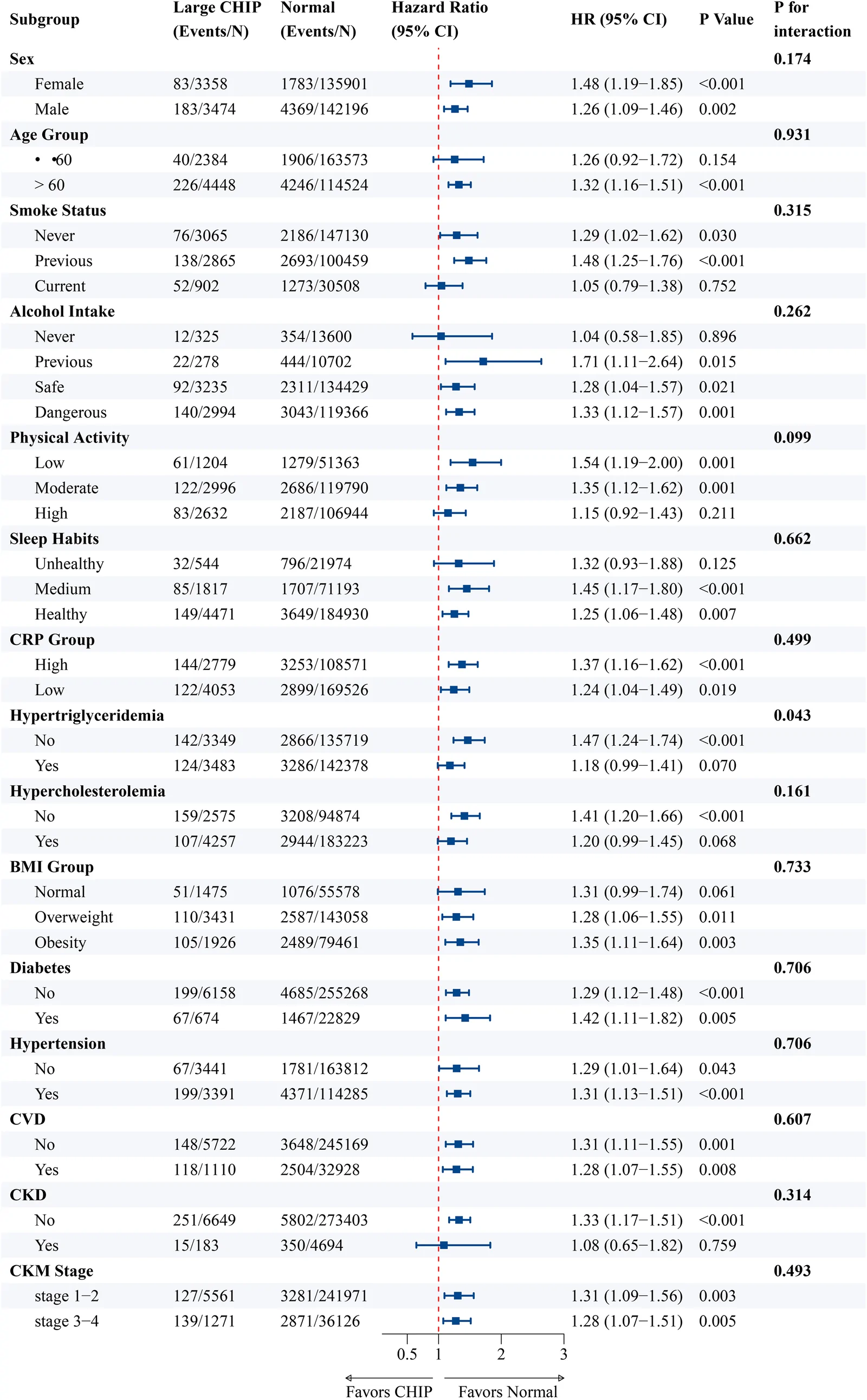
Subgroup analyses for the association between large CHIP and the risk of cardiovascular mortality. BMI, body mass index; CHIP, clonal hematopoiesis of indeterminate potential; CI, confidence interval; CKD, chronic kidney disease; CKM, cardiovascular-kidney-metabolic; CRP, C-reactive protein; CVD, cardiovascular disease; HR, hazard ratio.

### 3.4 Biological pathways and mechanisms

Within the proteomics sub-cohort (n = 31,702), 1,183 participants were identified as CHIP carriers [(with any CHIP (VAF ≥2%)], and a total of 903 cardiovascular death events occurred during the follow-up period. Following comprehensive covariate adjustment, **Figures 6A, B**; **Supplementary Tables S2**, **S3** detail the specific circulating proteins significantly associated with CHIP status and cardiovascular risk, respectively. By intersecting these two distinct proteomic signatures, we identified a consensus core of 58 overlapping proteins for downstream mechanistic exploration. **Figures 6C, D** delineate the results of the comprehensive GO and KEGG functional enrichment analyses. Specifically, **Figure 6C** highlights the top 10 most significant pathways ranked by their -log10 P-values, alongside the specific proteins driving these enrichments. In parallel, **Figure 6D** depicts the top 30 functional pathways strictly ordered by their overall enrichment degree. Integrative assessment of both statistical significance and enrichment magnitude revealed that the highest-ranking biological pathways were predominantly concentrated within distinct functional clusters. Specifically, the leading functional terms heavily implicated immune and inflammatory responses, featuring the regulation of general cell, leukocyte, and macrophage activation, alongside their corresponding negative regulatory processes. Furthermore, critical pathways governing tissue remodeling and structural development were also highly enriched, encompassing the secretory granule membrane, extracellular matrix disassembly, tertiary granule, outflow tract septum morphogenesis, and endodermal cell differentiation. To pinpoint the specific factors potentially underlying this risk, **Figure 6E** illustrates the precise proportion of the cardiovascular mortality association statistically mediated by each candidate protein. Consistent with the prominent immune signatures identified above, several key immune-regulatory receptors emerged as top mediators, with VSIG4 (33.1%), CD300C (29.2%), and THY1 (28.6%) accounting for substantial proportions of the excess risk. Alongside these immune effectors, Desmocollin-2 (DSC2), a critical structural component implicated in tissue remodeling, was identified as the single most prominent individual mediator, explaining 34.3% of the risk. The substantial effect sizes of these candidates strongly corroborate our pathway analysis, suggesting that CHIP drives adverse cardiovascular outcomes through a synergistic disruption of immune homeostasis and structural integrity. Of note, these proteomic and mediation analyses are exploratory in nature and should be interpreted as hypothesis-generating.

**Figure 6:**
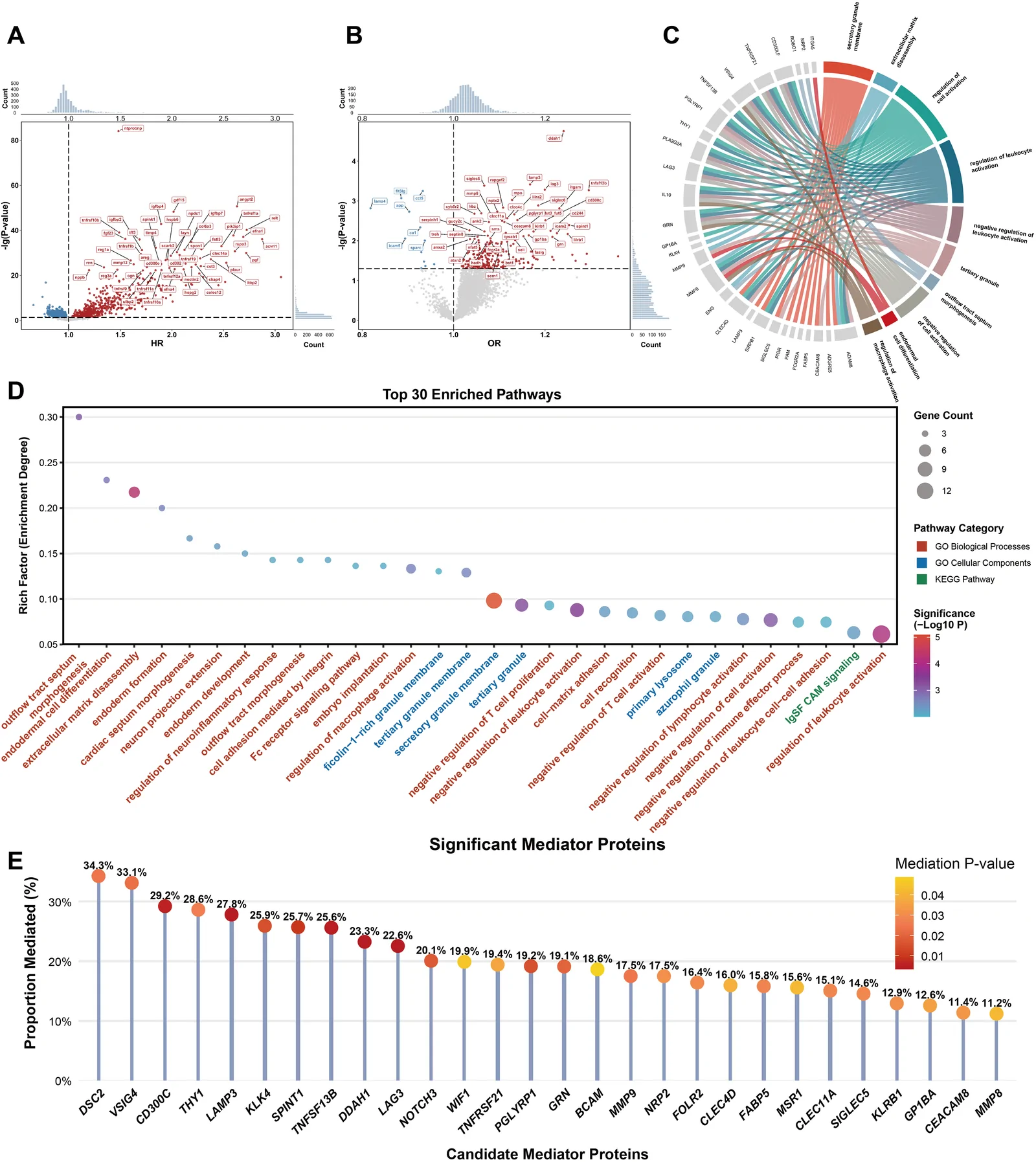
Proteomic analysis resolving the biological mechanisms linking CHIP status with increased cardiovascular mortality risk. **(A)** Volcano plot showing circulating proteins significantly associated with cardiovascular mortality risk. **(B)** Volcano plot showing proteins significantly associated with CHIP status. **(C)** Chord diagram highlighting the top 10 most significant GO and KEGG pathways ranked by -log10 P-values, illustrating the specific proteins driving these enrichments. **(D)** Bubble plot depicting the top 30 functional pathways strictly ordered by overall enrichment degree. **(E)** Mediation analysis results for incident cardiovascular mortality. CHIP, clonal hematopoiesis of indeterminate potential; GO, Gene Ontology; HR, hazard ratio; KEGG, Kyoto Encyclopedia of Genes and Genomes; OR, odds ratio.

## 4 Discussion

### 4.1 Principal findings

In this large-scale prospective cohort study leveraging whole-exome sequencing and long-term follow-up from the UKB, we established that the presence of CHIP, particularly large clones, is a robust and independent risk factor for cardiovascular mortality in the CKM syndrome population. Notably, harboring large CHIP was associated with a 28% increased risk of cardiovascular death, maintaining a remarkably stable prognostic penalty across both early and advanced disease stages. Furthermore, integrative proteomic analyses elucidated that this CHIP-driven excess risk is fundamentally mediated by profound immune dysregulation and compromised structural integrity. Interestingly, subsequent gene-specific analyses did not identify large clones of any single driver gene as an independent predictor of cardiovascular mortality. This lack of genotype-specific dominance suggests that clinical risk stratification strategies should prioritize the holistic assessment of overall CHIP burden rather than focusing extensively on identifying specific mutational profiles, positioning the aggregate CHIP status as a broadly applicable composite biomarker.

### 4.2 Comparison with other studies

While previous studies have well-established the link between CHIP and cardiovascular disease, our findings build upon and extend this understanding by systematically mapping the prognostic implications of CHIP across the entire continuum of cardiovascular-kidney-metabolic (CKM) disease states. By integrating CHIP into this novel multi-system framework, our study provides granular, stage-specific evidence that highlights.

Within the cardiovascular domain, a lot of literature has established CHIP as an independent risk determinant for disease, including ASCVD, heart failure, and arrhythmias (9, 22, 23). Shifting to prognostic outcomes, a previous analysis encompassing 13,129 patients with ASCVD from the UKB demonstrated that the presence of CHIP significantly increases the risk of mortality (12). Similarly, in a cohort of 8,612 patients with coronary artery disease, mutations in TET2, ASXL1, DNMT3A, and JAK2 were linked to elevated mortality, with TET2 carriers specifically exhibiting increased necrotic core volumes, intensified local inflammation, and compromised plaque stability (24). Furthermore, among 3,640 patients undergoing percutaneous coronary intervention, aggregate CHIP status emerged as an independent predictor of both all-cause and cardiovascular mortality (25). Beyond these cardiovascular manifestations, emerging evidence highlights the detrimental impact of CHIP variants on the renal function. Notably, carriers of non-DNMT3A myeloid CHIP exhibit a higher risk of incident CKD progression and faster renal function decline compared to non-carriers, positioning these specific clonal expansions as independent drivers of adverse renal events (10, 26, 27). The detrimental impact of CHIP is equally evident in metabolic dysregulation. Pooled multi-cohort analyses have revealed that CHIP status not only elevates the risk of incident type 2 diabetes mellitus (T2DM) but also serves as a strong risk factor for subsequent cardiovascular events among diabetic patients (11, 28). While prior studies generally evaluated cardiac, renal, and metabolic disorders separately, our study improves upon this approach by analyzing patients across the continuous CKM syndrome spectrum. We demonstrate that CHIP status served as an independent risk factor for cardiovascular mortality, irrespective of diverse baseline disease states and across both early and advanced CKM stages.

### 4.3 Mechanisms of CHIP-driven cardiovascular mortality

Gene-specific analyses in our cohort did not identify individual high-frequency CHIP drivers as independent predictors of cardiovascular mortality. This lack of significance is likely driven by statistical power limitations and the competing risks inherent in the diverse etiologies comprising cardiovascular death. Nevertheless, the overarching cardiovascular risk conferred by CHIP is well-supported by robust mechanistic evidence. Rather than acting through a singular pathway, diverse CHIP mutations drive cardiovascular mortality through mutation-specific trajectories that converge on shared adverse phenotypic outcomes across different cardiac tissues.

In the setting of atherosclerotic cardiovascular disease (ASCVD), various CHIP mutations (e.g., TET2, DNMT3A, ASXL1, TP53, and JAK2) predominantly accelerate atherogenesis and promote plaque instability. This is achieved via hyperactivation of myeloid innate immunity, exacerbated foam cell formation, and accelerated local macrophage proliferation, ultimately driving plaque vulnerability and erosion (24, 29–31). Beyond the vasculature, the adverse associations of CHIP extend to myocardial remodeling and HF. Specifically, TET2 mutations exacerbate HF, particularly HF with preserved ejection fraction (HFpEF), as deficient macrophages drive diastolic dysfunction and cardiac fibrosis via sustained NLRP3 inflammasome activation and IL-1β production (32–34). DNMT3A variants induce fibrosis through distinct intercellular crosstalk, where mutated macrophages secrete HB-EGF to activate resident fibroblasts and mutated monocytes mediate aberrant T-cell interactions (35, 36). Similarly, in atrial fibrillation (AF) and other arrhythmias, the local pro-inflammatory milieu generated by CHIP variants directly precipitates electrical instability (e.g., aberrant calcium leak) while simultaneously exacerbating interstitial fibrosis (22, 37, 38).

Despite the phenotypic differences among ASCVD, HF, and AF, previous studies indicate that their underlying mechanisms converge on two core processes: systemic immune dysregulation and aberrant structural tissue remodeling. This consensus aligns with our proteomic mediation findings. Consistent with the immune-driven pathology reported in experimental models, our functional enrichment analysis demonstrated significant dysregulation in leukocyte and macrophage activation pathways. Furthermore, the mediation analysis identified specific immune-regulatory receptors, namely VSIG4 (33.1%), CD300C (29.2%), and THY1 (28.6%), as major candidate mediators of CHIP-associated mortality (39–42). Because these circulating proteins regulate macrophage homeostasis and T-cell interactions, these results provide exploratory human epidemiological evidence linking clonal myeloid mutations to systemic immune activation. Addressing the shared fibrotic and structural consequences, such as plaque vulnerability and myocardial fibrosis, our study identified Desmocollin-2 (DSC2) as prominent candidate mediator, which accounted for 34.3% of the excess risk. As a structural component of desmosomes involved in tissue integrity, these exploratory mediation estimates suggest a potential mechanistic link between CHIP and adverse cardiovascular structural remodeling rather than providing definitive causal confirmation (43, 44).

In summary, CHIP independently increases the risk of cardiovascular mortality. Rather than acting through isolated, disease-specific pathways, CHIP may serve as an upstream factor of both systemic inflammation and structural remodeling, a dual pathway hypothesis supported by our exploratory proteomic profiling that warrants future mechanistic validation.

### 4.4 Clinical translation

CKM syndrome is an emerging concept defined as a systemic disorder driven by pathophysiological interactions among obesity, T2DM, CKD, and CVD. According to recent NHANES data, nearly 90% of US adults are classified within CKM stages 1 to 4 (2). Our study reveals that the adverse associations of CHIP are not confined to a single pathology; rather, they span a vast disease continuum, from the presence of initial metabolic risk factors to established CKM conditions. Notably, while the relative risk (hazard ratios) conferred by large CHIP remained consistent across early and advanced CKM stages, this pattern must be interpreted through the lens of absolute risk. Because baseline cardiovascular mortality in advanced CKM (Stages 3-4) is substantially higher than in early CKM (Stages 1-2), a comparable relative hazard translates into a much larger absolute burden of excess cardiovascular deaths in later stages. This demonstrates a robust positive additive interaction between CHIP and advancing CKM severity, indicating that the absolute clinical toll of CHIP is amplified progressively as the metabolic substrate becomes more fragile. As a potential contributor to systemic inflammation and adverse outcomes, CHIP may help identify a subgroup at higher risk across the CKM continuum, but its clinical utility requires further prospective validation. Furthermore, individuals in CKM Stage 0 were excluded from the primary survival analysis. Because CKM stage 0 represents individuals without identifiable CKM risk factors or disease, we restricted the primary analysis to CKM stages 1–4 to align the analytic population with the objective of evaluating CHIP across the CKM disease continuum. Focusing on Stages 1–4 allowed us to accurately characterize CHIP’s prognostic impact within the actively progressing CKM pathological continuum.

Currently, there are no official guidelines for screening clonal hematopoiesis (CH) sequence variants. Existing population-based cardiovascular risk prediction models do not incorporate CH, nor are there targeted therapies specifically designed for CH-associated cardiovascular risk. Although gene-specific analyses showed that individual driver mutations (such as DNMT3A) were largely attenuated after demographic adjustment, likely reflecting reduced statistical power in smaller subgroups as well as distinct biological differences in pathogenicity, aggregate large CHIP demonstrated consistent, independent prognostic value. Translating these holistic markers into clinical workflows presents inherent challenges given these gene-specific variations and the current scarcity of personalized risk-tailoring strategies. However, because CH is frequently an incidental finding during DNA sequencing performed for other clinical indications, retrospectively determining CHIP status in populations with existing whole-genome sequencing data may hold significant clinical value (45, 46). However, it remains uncertain whether integrating CHIP into routine cardiovascular mortality screening will incrementally improve the predictive performance of traditional risk assessment tools. Furthermore, given the dynamic nature of CHIP development, longitudinal sampling to evaluate clonal evolution could provide additional prognostic insights. Given the potential for chance findings due to multiple subgroup comparisons, this isolated nominal interaction should be interpreted with caution and warrants validation in future studies.

### 4.5 Limitation

First, while the use of population-scale WES provides high fidelity for detecting candidate gene mutations, it possesses limited sensitivity for detecting smaller clones with a low VAF (e.g., VAF <5%) compared to targeted deep sequencing (22). Furthermore, we employed a stringent filtering protocol to minimize false-positive CHIP calls. While ensuring high specificity, this approach likely resulted in the under-detection of individuals with smaller clones, thereby underestimating the true prevalence of CHIP (14, 22). Importantly, any such non-differential misclassification would bias our observed associations toward the null, suggesting our risk estimates are ultimately conservative. Second, CHIP status was ascertained exclusively at baseline. This single time-point assessment precludes the evaluation of dynamic clonal evolution and fails to capture individuals who may have acquired CHIP mutations during the follow-up period. Third, outcome ascertainment relied primarily on diagnostic codes (e.g., ICD codes) derived from linked electronic health records, which carries an inherent risk of outcome misclassification, although previous validations within this cohort have consistently demonstrated high diagnostic accuracy. Fourth, the study cohort consists predominantly of middle-aged to older individuals of European ancestry and is subject to a well-documented healthy volunteer bias (47). These demographic characteristics limit the generalizability of our findings to younger populations, diverse racial and ethnic groups, or populations with different baseline cardiometabolic risk profiles. Additionally, the absence of an independent external validation cohort necessitates cautious interpretation, and future studies incorporating multi-center, diverse cohorts are warranted to validate and extend our current findings. Fifth, the exclusion of participants with missing or indeterminate CKM staging may have introduced selection bias and limits the generalizability of our findings to individuals with evaluable CKM staging. Sixth, despite the large overall sample size, statistical power remained limited for evaluating ultra-rare CHIP driver mutations, exploring variant-specific interactions, or conducting granular subgroup analyses across distinct clinical phenotypes. Seventh, given the observational nature of this cohort study, the possibility of unmeasured or residual confounding underlying the associations between CHIP and adverse clinical outcomes cannot be definitively excluded. Finally, because this study relies on observational longitudinal data from the UK Biobank cohort, the findings represent associations between CHIP status, CKM syndrome stages, and cardiovascular mortality. Therefore, definitive causal relationships cannot be established, and future experimental or Mendelian randomization studies are warranted to elucidate the direct underlying mechanisms.

## 5 Conclusion

The prevalence of CHIP escalates in tandem with advancing CKM stages. Within the CKM syndrome population, harboring CHIP mutations emerges as a robust, independent risk factor for cardiovascular mortality. Crucially, this adverse association spans the entire disease continuum, remaining significant in both early and advanced stages, irrespective of the presence of established cardiovascular, kidney, or metabolic diseases. Mechanistically, CHIP is associated with an exacerbated cardiovascular mortality risk, potentially via pathways involving systemic inflammation, immune dysregulation, and aberrant tissue remodeling. Consequently, profiling CHIP status may help refine risk stratification across the CKM spectrum, but its clinical utility requires further prospective validation before it can inform management.

## Data Availability

Publicly available datasets were analyzed in this study. This data can be found here: https://www.ukbiobank.ac.uk/.
