# Supplementary material for: Association of clonal hematopoiesis of indeterminate potential with cardiovascular mortality in cardiovascular-kidney-metabolic syndrome: a large prospective cohort study
Source: Front Endocrinol (Lausanne). 2026 Oct 7;17:1910361. doi: 10.3389/fendo.2026.1910361 (PMC13684865; doi:10.3389/fendo.2026.1910361)
Supplement: Supplementary file 1 [file DataSheet1.docx]

Figure 1. Distribution of Continuous Covariates Before and After Missing Value Imputation

Figure 2. Proportional Distribution of Categorical Covariates Before and After Missing Value Imputation

Figure 3. Association of CHIP clone size with cardiovascular mortality risk across CKM stages using a competing risk model.

Figure 4. Association of CHIP clone size with cardiovascular mortality risk across CKM stages using an expanded outcome definition.

Figure 5. Association of CHIP clone size with cardiovascular mortality risk across CKM stages after excluding early events.
