# Supplementary material for: Association of clonal hematopoiesis of indeterminate potential with cardiovascular mortality in cardiovascular-kidney-metabolic syndrome: a large prospective cohort study
Source: Front Endocrinol (Lausanne). 2026 Oct 7;17:1910361. doi: 10.3389/fendo.2026.1910361 (PMC13684865; doi:10.3389/fendo.2026.1910361)
Supplement: Supplementary file 2 [file Table1.docx]

1. Assessment of CHIP Exposures
   (1) Whole-Exome Sequencing and Variant Calling

As described in the main text, CHIP status and related variant characteristics (UK Biobank Data-Fields 30105, 30106, and 30107) were derived from whole-exome sequencing (WES) of peripheral blood samples. Sequencing was performed at the Regeneron Genetics Center (Tarrytown, NY) using the Illumina NovaSeq 6000 platform. Somatic variant calling was performed with Mutect2 in the Genome Analysis Toolkit (GATK). Candidate CHIP variants were restricted to the canonical CHIP driver-variant panel used in the previously validated UK Biobank pipeline (13). This panel comprises recurrent missense, indel, truncating, and splice-site variants in CHIP- and myeloid malignancy-associated driver genes, and the full gene list is provided in Supplementary Table S1. Synonymous and other noncoding variants were not retained. For U2AF1, hotspot variants were identified using the custom pileup-based procedure described in the validated pipeline because standard Mutect2 calling is unreliable at the duplicated locus on GRCh38.

(2) Quality Control and Variant Filtering

To ensure high-confidence somatic variant identification and minimize false-positive calls, variants were retained for downstream analyses only if they satisfied all of the following criteria: (i) total sequencing depth of at least 20 reads; (ii) alternate-allele depth of at least 5 reads, consistent with the optimized UK Biobank threshold; and (iii) support on both forward and reverse sequencing strands. Putative variants were annotated before downstream filtering, and sequencing artifacts and germline variants were removed using the previously established curation workflow (13). Briefly, recurrent candidate variants were screened using population-level patterns, and variants that were not associated with age or the TERT promoter variant rs7705526 at P < 0.10 were removed as likely sequencing artifacts. Likely germline variants were further evaluated using a binomial test of allelic balance, and variants failing the test at P < 0.01 were removed unless they represented established CHIP hotspots. Somatic mosaic CHIP calls were retained only when they passed these filters. Because CHIP ascertainment was based on WES SNV/indel calls, copy-number alterations and other structural mosaic events were not included in the operational CHIP definition.

(3) Definition of CHIP and CHIP Subtypes

CHIP exposures were strictly defined based on variant allele frequency (VAF) thresholds. Overall Any CHIP was defined as the presence of at least one somatic mutation with a VAF > 2%, whereas overall Large CHIP was defined as the presence of a mutation with a VAF > 10%.

For gene-specific analyses (e.g., focusing on DNMT3A, TET2, and ASXL1), equivalent VAF criteria were applied to individual driver genes. A gene-specific Any CHIP or gene-specific Large CHIP was identified when the VAF for that particular gene was > 2% or > 10%, respectively.

To systematically isolate the distinct prognostic impact of specific driver genes and prevent statistical confounding, mutually exclusive classification frameworks were constructed. For instance, when evaluating the specific effect of DNMT3A, participants were stratified into specific categories based on the respective VAF thresholds (2% or 10%):

(i) DNMT3A CHIP / DNMT3A Large CHIP: Individuals meeting the VAF threshold specifically for the DNMT3A gene;

(ii) Non-DNMT3A Any CHIP / Non-DNMT3A Large CHIP: Individuals meeting the overarching criteria for overall Any CHIP or Large CHIP, but explicitly lacking the DNMT3A mutation at the corresponding threshold;

(iii) No CHIP: Individuals with an absolute absence of any detectable mutations (VAF = 0), serving as the universal reference group across all models.

2. Missing Data Imputation Strategy

To minimize bias and maintain statistical power, missing data were imputed using the missRanger package in R, which integrates the Multivariate Imputation by Chained Equations (MICE) framework with the fast random forest implementation, ranger.

The imputation algorithm was configured to balance predictive performance with computational efficiency. Specifically, the following parameters were implemented:

(1) Iterative Process: A maximum of 5 iterations was set to achieve convergence across the variables.
(2) Random Forest Configuration: Each forest consisted of 1000 trees. To optimize processing speed, a sample fraction of 0.5 was utilized for growing each tree, and the minimum node size was fixed at 10.
(3) Predictive Mean Matching (PMM): To ensure that imputed values remained within the biologically and physically plausible range of the observed data and to preserve the original data distributions, PMM was applied with a matching pool of k = 5 donors.

3. Definition of Cardiovascular-Kidney-Metabolic syndrome stage:
   (1) CKM Stage 0: Individuals with normal BMI and waist circumference, normoglycemia, normotension, a normal lipid profile, and no evidence of CKD or subclinical or clinical CVD.
   (2) CKM Stage 1: Individuals with overweight/obesity, abdominal obesity, or dysfunctional adipose tissue, without the presence of other metabolic risk factors or CKD
       1. BMI ≥25 kg/m² (or ≥23 kg/m² if Asian ancestry),
       2. Waist circumference ≥88/102 cm in women/men (or if Asian ancestry ≥80/90 cm in women/men), or
       3. Fasting blood glucose ≥100–124 mg/dL or HbA1c between 5.7% and 6.4%.
   (3) CKM Stage 2: Individuals with metabolic risk factors (including hypertriglyceridemia [≥135 mg/dL], hypertension, metabolic syndrome, or diabetes) or chronic kidney disease (CKD).
   (4) CKM Stage 3: Individuals with subclinical atherosclerotic cardiovascular disease (ASCVD) or subclinical heart failure (HF) who also have excess/dysfunctional adiposity, other metabolic risk factors, or CKD. This stage also encompasses risk equivalents of subclinical CVD, which include:
       1. Very high-risk CKD (defined as stage G4 or G5 CKD, or very high risk per the KDIGO classification).
       2. High predicted 10-year CVD risk (PREVENT risk score > 0.2).
   (5) CKM Stage 4: Individuals with established clinical cardiovascular disease (such as coronary heart disease, HF, stroke, peripheral artery disease, or atrial fibrillation) among those with excess/dysfunctional adiposity, other CKM risk factors, or CKD.
4. Definition of variables
   (1) Diabetes status

| Category | Definition Criteria | Data Fields |
| --- | --- | --- |
| Hospital Inpatient Records | Diagnosis of diabetes based on ICD-10 codes (E10–E14) or ICD-9 codes (250.0–250.9) in hospital inpatient records, with a diagnosis date prior to the date of attending the assessment centre. | 53, 130706, 130708, 130710, 130712, 130714, [41271](https://biobank.ndph.ox.ac.uk/showcase/field.cgi?id=41271), [41281](https://biobank.ndph.ox.ac.uk/showcase/field.cgi?id=41281) |
| Self-reported History | Self-reported diagnosis of diabetes by a doctor during the touchscreen questionnaire or verbal interview at baseline. | 2976, 20002, 2443 |
| Medication Use | Self-reported use of glucose-lowering medications (e.g., insulin, metformin) at baseline. | 20003, 6177, 6153 |
| Fasting Plasma Glucose | Fasting plasma glucose level ≥ 7.0 mmol/L. | 74, 30740 |
| Random Plasma Glucose | Random plasma glucose level ≥ 11.1 mmol/L. | 74, 30740 |
| HbA1c | Glycated hemoglobin (HbA1c) level ≥ 6.5% (48 mmol/mol). | 30750 |

(2) Cardiovascular Disease

| Disease | Category | Definition Criteria | Data Fields |
| --- | --- | --- | --- |
| IHD | Hospital Records & Algorithmic Definitions & self-reported medical history | CAD was ascertained through hospital admission records using ICD-9 codes 410–414 and ICD-10 codes I20–I25, combined with algorithmically defined myocardial infarction events provided by UK Biobank. | 53, [41271](https://biobank.ndph.ox.ac.uk/showcase/field.cgi?id=41271), [41281](https://biobank.ndph.ox.ac.uk/showcase/field.cgi?id=41281), 42000, 42002, 42004, 131296, 131298, 131300, 131302, 131304, 131306 |
| Stroke | Hospital Records & Algorithmic Definitions & self-reported medical history | Stroke was ascertained through hospital admission records using ICD-9 codes 430–435 and ICD-10 codes I60–I64, combined with algorithmically defined stroke events provided by UK Biobank. | 53, [41271](https://biobank.ndph.ox.ac.uk/showcase/field.cgi?id=41271), [41281](https://biobank.ndph.ox.ac.uk/showcase/field.cgi?id=41281), 42006, 42008, 42010, 42012, 131360, 131362, 131364, 131366, 131368 |
| Atrial fibrillation | Hospital Records & self-reported medical history | Atrial fibrillation was ascertained through hospital admission records using ICD-9 codes 4273 and ICD-10 codes I48 | 53, [41271](https://biobank.ndph.ox.ac.uk/showcase/field.cgi?id=41271), [41281](https://biobank.ndph.ox.ac.uk/showcase/field.cgi?id=41281), 131350 |
| Heart failure | Hospital Records & self-reported medical history | Heart failure was ascertained through hospital admission records using ICD-9 codes 428 and ICD-10 codes I50 | 53, [41271](https://biobank.ndph.ox.ac.uk/showcase/field.cgi?id=41271), [41281](https://biobank.ndph.ox.ac.uk/showcase/field.cgi?id=41281), 131354 |

(3) Chronic kidney disease

| Category | Definition Criteria | Data Fields |
| --- | --- | --- |
| Estimated GFR (eGFR) | Estimated glomerular filtration rate (eGFR) < 60 mL/min/1.73 m^2^ at baseline, calculated using the CKD-EPI equation based on serum creatinine and/or cystatin C. | 31, 30700, 21003 |
| Hospital Records & Algorithmic Definitions | Diagnosis of CKD based on ICD-10 code N18 or ICD-9 code 585 in hospital inpatient records, or identified as having end-stage renal disease based on UK Biobank algorithmically defined outcomes prior to recruitment. | 53, 132032, 42026, [41271](https://biobank.ndph.ox.ac.uk/showcase/field.cgi?id=41271), [41281](https://biobank.ndph.ox.ac.uk/showcase/field.cgi?id=41281) |

(4) Socioeconomic factors
    1. Townsend Deprivation Index (Field 22189): Socioeconomic disadvantage was evaluated via the TDI, a continuous metric derived from the preceding national census. This score integrates four area-level domains—unemployment, household overcrowding, and lack of home or vehicle ownership—and was assigned based on the participant's residential postcode at baseline.
    2. Employment status (Field 6142): Occupational status was grouped into three distinct categories: employed (encompassing salaried workers, the self-employed, and students), retired, and unemployed (which included individuals unable to work or caring for a home/family). Uninformative responses, such as "prefer not to answer" or "none of the above," were coded as missing (NA).
    3. Educational attainment (Field 6138): Education level was stratified into four tiers: college degree (university, NVQ, HND, HNC, or equivalent), high school (A/AS levels or vocational qualifications), middle school (O levels, GCSEs, or CSEs), and primary education or less ("none of the above").
    4. Annual household income (Field 738): Self-reported pre-tax household earnings were divided into five specific brackets: <£18,000, £18,000–£30,999, £31,000–£51,999, £52,000–£100,000, and >£100,000. Responses indicating uncertainty ("do not know") or refusal to answer were excluded as missing data.
(5) behavioral factors
    1. Smoking status (Field 20116): Based on baseline questionnaire data, individuals were stratified into current, previous, or never smokers. Uninformative answers (e.g., "prefer not to answer") and missing records were excluded as missing values (NA).
    2. Drinking status and alcohol intake (Fields 20117, 1568, 4407, 1578, 4418, 1588, 4429, 1598, 4440, 1608, 4451, 5364, 4462): Alcohol consumption was assessed by integrating self-reported drinking history and a detailed quantification of weekly intake. For current drinkers, total weekly alcohol units were calculated by summing the consumption across various beverage types (Field 1568–5364 for weekly and 4407–4462 for monthly intake). Monthly frequencies were converted to weekly intake by dividing by 4.3.

To ensure standardized quantification, one alcohol unit was defined as 10 mL (8 g) of pure ethanol. Based on UK Biobank’s standard conversion factors, specific unit values were assigned to standard measures of different beverages:

- 1.5 units per glass of red wine, white wine, or champagne;
- 2.8 units per pint of beer or cider;
- 1.0 unit per standard measure of spirits or fortified wine (e.g., sherry or port);
- 1.5 units per measure of other alcoholic beverages.

Participants were subsequently categorized into four distinct groups according to their drinking history and the UK Chief Medical Officers' low-risk drinking guidelines:

- Never drinkers: Participants with no history of alcohol consumption;
- Previous drinkers: Those who had consumed alcohol in the past but not currently;
- Safe drinkers: Current drinkers consuming 0–14 units per week;
- Dangerous drinkers: Current drinkers consuming more than 14 units per week.

Participants who responded "Do not know," "Prefer not to answer," or had missing data for these fields were treated as missing values (NA).

1. Physical activity level (Fields 22038, 22039): Exercise volume was evaluated via Metabolic Equivalent Task (MET) scores. A composite weekly MET-minutes metric was computed using the formula: moderate METs + (vigorous METs × 2). Participants were subsequently dichotomized into Healthy (≥150 MET-minutes/week) and Unhealthy (<150 MET-minutes/week) activity groups.
2. Sleep status (Field 1160): Self-reported daily sleep duration was utilized to define sleep health. A Healthy sleep pattern was characterized by 7 to 9 hours of sleep per 24-hour cycle, whereas individuals reporting <7 hours or ≥9 hours were deemed to have an Unhealthy sleep status.
